# Outcome of prenatal diagnosis of clubfoot: a single institution experience

**DOI:** 10.2144/fsoa-2021-0106

**Published:** 2021-12-01

**Authors:** Wael Abdallah, Malek Nassar

**Affiliations:** 1 Department of Gynecology & Obstetrics, Hôtel-Dieu de France University Hospital, Saint Joseph University, Beirut, Lebanon; 2 Clinique de Diagnostic Prénatal, Zalka, 1202, Lebanon

**Keywords:** clubfoot, diagnostic accuracy, fetal outcomes, prenatal diagnosis

## Abstract

**Aim:**

To assess the accuracy of antenatal diagnosis of clubfoot (CF), risk factors and outcomes in postnatal.

**Patients & methods:**

Maternal characteristics, sonographic signs and postnatal outcomes were evaluated in 60 patients with a prenatal diagnosis of CF between 2007 and 2020.

**Results:**

The rate of antenatal diagnosis of CF was 3.72/1000 live births. The false-positive rate was 6.67%. 66.7% of fetuses had bilateral CF and 33.3% had unilateral CF; 58.3% were isolated and 41.7% were complex; 58.3% were males and 41.7% were female; 16.7% were multiple pregnancies and 10% were cases of consanguinity.

**Conclusion:**

The accuracy of the diagnosis of CF depends on the operator’s skills. A significant relationship is demonstrated between the interruption of pregnancy, consanguinity, laterality and complexity.

## Lay abstract

CF is one of the most common fetal anomalies detected antenatally on ultrasound, occurring in approximately 1/1000 live births. We present our single institution experience with the antenatal diagnosis of CF. Over 60 cases, the rate of prenatal diagnosis of CF was 3.72/1000 live births; of which 33.3% had unilateral CF; 58.3% were isolated and 41.7% were complex; 58.3% were males and 41.7% were female; 16.7% were multiple pregnancies and 10% were cases of consanguinity.

Clubfoot (CF), known as congenital talipes equinovirus, is one of the most common fetal anomalies detected prenatally on ultrasound, occurring in approximately 1/1000 live births [1]. It is a combination of four elements: metatarsus adductus, cavus foot, heel varus and equinus (Figure 1) [2]. As a result, the fetal foot would present as fixed in supination, adduction, varus or valgus with a subluxation of talocalcaneoclavicular joint [3]. It can be unilateral or bilateral, isolated or complex – associated with other malformations and structural defects [4]. Moreover, it can result from a congenital anomaly affecting the soft and neurological tissues, muscles and bone; or from mechanical factors reducing fetal mobility, such as breech presentation, uterine malformation, oligohydramnios and multiple gestations [5]. Thus, international guidelines recommend identifying both legs and feet in the second-trimester fetal ultrasound scan, while the description of their position and relationship is not obligatory [6]. Despite the improvement of prenatal scanning for fetal anomalies, the false-positive rate (FPR) of the diagnosis of isolated CF ranges from 10 to 40% [7,8]. The observation of the tibia and fibula in the same plane as the sole of the foot is essential to be able to diagnose CF. Additionally, searching for possible associated abnormalities, or skeletal and neurological malformations is necessary to identify ‘complex CF’ [9]. Of concern, 13% of cases with CF had associated abnormalities not diagnosed prenatally [7]. Furthermore, 30% of complex CF can be associated with chromosomal aberrations [4]. Besides, clinically significant chromosomal microarray analysis (CMA) results are detected in approximately 4% of isolated CF [5]. Otherwise, karyotypic evaluation is still controversial in isolated CF, but reviewing the literature may suggest that it might seem beneficial [7,10]. The prenatal diagnosis of CF is essential and should be followed by counseling to provide the child’s parents with information about the diagnosis and the outcomes. Therapeutic options include conservative treatment like serial casting or surgery.

**Figure 1.**
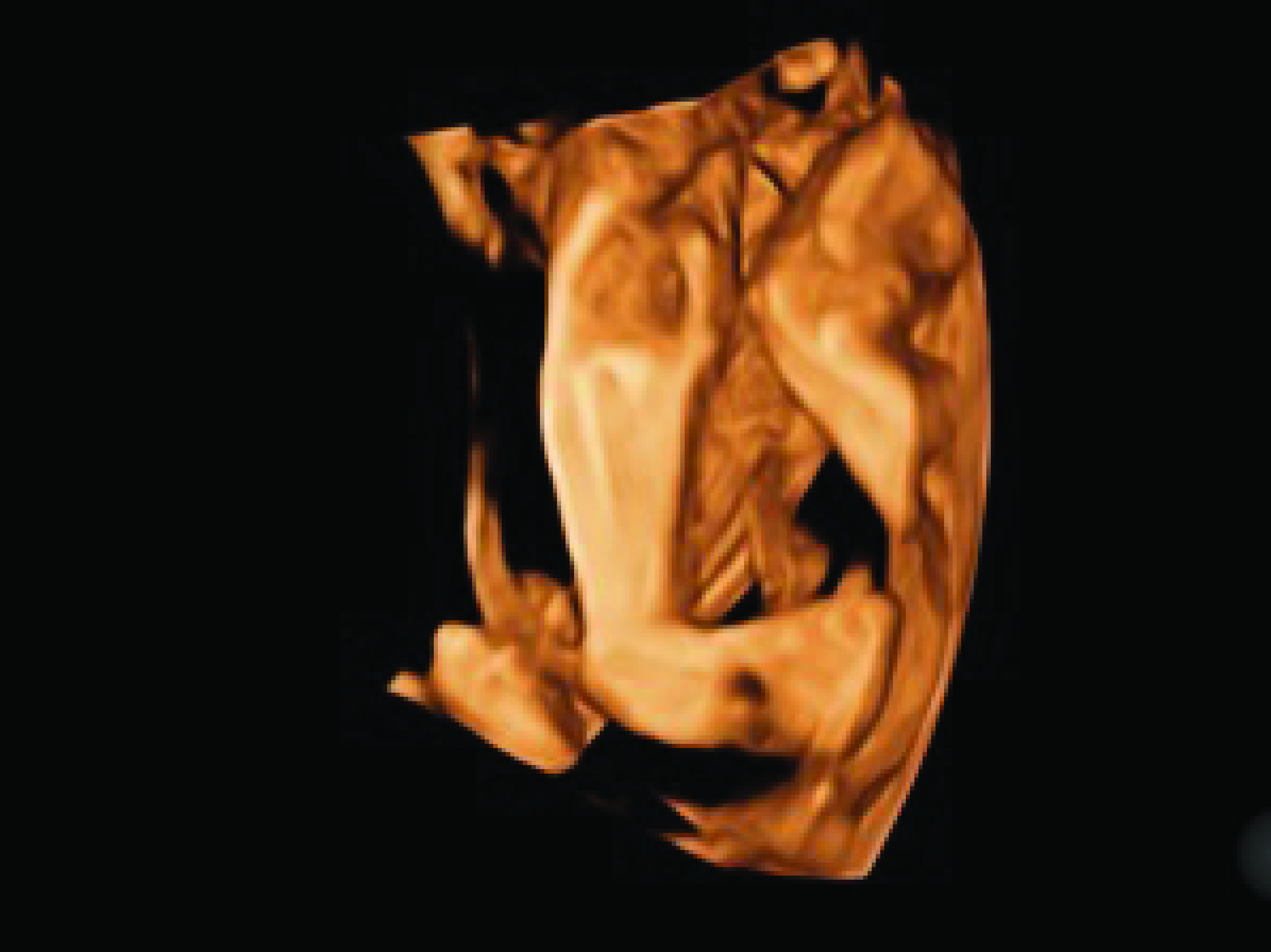
Sonographic image of clubfoot.

The aim of this study was to investigate the accuracy of CF diagnosis in our center, the presence of chromosomal abnormalities, the rate of associated findings and the overall outcome in newborns prenatally diagnosed with CF.

## Methods

A retrospective analysis was made for all pregnant patients diagnosed prenatally with congenital fetal CF referred to our institution Clinique de Diagnostic Prenatal between 2007 and 2020. The diagnosis was identified by 2D and 3D imaging using a volumetric US probe (GE Voluson E6, WI, USA), and was performed by the same expert operator. When the plantar face of the fetal foot was present in the same plane as that of the tibia and fibula, the diagnosis of fetal CF was adopted. All cases underwent detailed sonographic scanning to detect associated malformations. The evaluation was followed by a control ultrasound in case the operator felt the diagnosis was not clear enough. In addition, invasive diagnosis by amniocentesis was suggested to all parents while informing them about the risks of this procedure. We classify CF as isolated if no other anomalies were detected, and as complex if any anomaly or defect was detected. We identified a termination of pregnancy as voluntary, medically or spontaneously ending of pregnancy. Genetic syndromes or malformations diagnosed after birth were also reported. Furthermore, postnatal follow up was obtained and the type of treatment (conservative or surgical) was also reported. The diagnosis of CF in postnatal was confirmed or declined in all cases by examination of pediatric orthopedic surgeon. In the case of stillbirth or intrauterine fetal demise, fetuses were sent to pathology examinations.

Statistical analysis was provided with Statistical Package for the Social Sciences software, SPSS 25. Normality of distribution was studied using skewness and kurtosis. Normal continuous variables were analyzed using student *t*-test. Other tests were helpful in this study, such as the chi-square test. A probability value p-value < 0.05 was considered to be statistically significant.

## Results

During the study period, 60 prenatal diagnoses of CF were identified among 16,096 live births, yielding a rate of 3.72 per 1000 live births. Forty (66.7%) fetuses had bilateral CF and 20 (42.33%) had unilateral CF, 35 (58.3%) had isolated CF and 25 (58.33%) had complex CF, 35 (58.3%) were males and 25 (58.33%) were females, ten (16.7%) were multiple pregnancies, and we did not have any case where both twins were affected. The majority of the fetuses were cephalic at the moment of the diagnosis (61.7%). Besides, the range of maternal age was between 22 and 41 years old with a median of 32. The median of the gravida was 2, with a range of 1–10. We reported six (10%) couples with consanguinity. Fifty-seven (95%) of the mothers were nonsmokers. Fifty-seven (95%) of the patients were diagnosed in the second trimester while three of the patients were diagnosed in the third trimester. The median of gestational age at the time of diagnosis was 22^+3^ weeks, the most precocious one was at 14^+3^ weeks. Of the 60 patients, 20 (33.3%) underwent an amniocentesis: 16 karyotypes were normal, while one revealed a translocation 5–7 with isolated CF, one showed a Turner syndrome with complex CF and another one revealed Trisomy 13 with also complex CF. Twelve of the 20 cases (60%) who underwent amniocentesis were diagnosed with isolated CF. Of the 60 fetuses included in the study, a diagnosis of CF was confirmed in 56 neonates, with a FPR of 6.67%. One prenatal diagnosis of complex CF associated with clubhand turned out to be isolated CF postnatally. Additionally, the same case was diagnosed with unilateral CF postnatally, while he was prenatally labeled as bilateral. Table 1 summarizes the association between the accuracy of the antenatal diagnosis of CF and several clinical and obstetrical characteristics of the population. The rate of false positive is independent of maternal age, gestational age at the moment of the diagnosis, fetal gender and presentation, single or multiple pregnancies, and the complexity and the laterality of CF. On the other hand, 14 (23.3%) of the cases ended with medical interruption of pregnancy and nine (15%) ended with stillbirth. Table 2 shows the association between pregnancy outcomes and multiple maternal and fetal characteristics. We conclude consanguinity, laterality complexity of CF are risk factors for pregnancy termination. Moreover, the frequency of bilateral isolated CF was 30%, with a significant association between the laterality and the complexity of CF (p = 0.003). Two cases presented an oligohydramnios, one associated with hydramnios, while all the other cases had normal amniotic fluid index. We did not find any association between the amniotic fluid level and the diagnosis of CF. On the other hand, four (12.5%) infants profited from exclusive physical therapy, while 17 (53%) needed Ponseti method, and exclusive progressive casting was favorable in nine cases (29%). Surgical treatment was not needed in any case. Furthermore, we did not find any significant association between the Ponseti method, the complexity (p = 0.17) and the laterality of CF (p = 0.98), the parity (p = 0.28) and fetal presentation (p = 0.05). Of concern, all children with CF were walking and had an average outcome score of ‘very good’ to ‘excellent.’

**Table 1.** The accuracy of the prenatal diagnosis of clubfoot in function of several maternal and fetal characteristics with p-value (A) and odds ratio (B).

A			
Variable	Confirmed CF (56)	No CF (4)	p-value
Maternal age (year)	31.68 ± 5.03	34 ± 2.16	0.12
GA at US evaluation (week)	22.35 ± 3.4	23.5 ± 2.6	0.45
Unilateral	17 (30.4%)	3 (75%)	0.06
Bilateral	39 (69.6%)	1 (25%)	0.06
Isolated	31 (55.4%)	4 (100%)	0.08
Complex	25 (44.6%)	0 (0 %)	0.08
Female	23 (41.1%)	2 (50%)	0.72
Male	33 (58.9%)	2 (50%)	0.72
Breech	16 (28.6%)	1 (25%)	0.75
Cephalic	34 (60.7%)	3 (75%)	0.75
Transverse	6 (10.7%)	0 (0%)	0.75
Single pregnancy	47 (83.9%)	3 (75%)	0.64
Multiple pregnancy	9 (16.1%)	1 (25%)	0.64

** B**			
	Anscombe-Haldane corrected OR	95% lower limit	95% upper limit
Bilateral CF	0.15	0.01	1.5
Multiple pregnancy	1.74	0.16	18.68
Complex CF	0.14	0.01	2.67
Multiple pregnancy	0.65	0.06	6.67
Female gender	0.7	0.09	5.31
Breech presentation	0.83	0.08	8.62
Cephalic presentation	1.94	0.19	19.87
Transverse presentation	0.86	0.04	17.93

CF: Clubfoot; GA: Gestational age; OR: Odds ratio; US: Ultrasound.

**Table 2.** The association between the outcomes of the pregnancy and different variables.

Variable	Normal evolution (37)	Medical interruption (14)	Stillbirth (9)	p-value
Consanguinity	37 (100%)	11 (78.6%)	6 (66.7%)	**0.003**
No consanguinity	0 (0%)	3 (21.4%)	3 (33.3%)	
Isolated	33 (89.2%)	1 (7.1%)	1 (11.1%)	**<0.001**
Complex	4 (10.8%)	13 (92.9 %)	8 (88.9%)	
Unilateral	18 (48.6%)	1 (7.1%)	1 (11.1%)	**0.006**
Bilateral	19 (51.4%)	13 (92.9%)	8 (88.9%)	

p-values < 0.05 are considered to be statistically significant and are in boldface.

## Discussion

The prenatal diagnosis of CF is essential and should be followed by parents counseling to inform them about the outcomes of CF. The prevalence of congenital CF varies between 1–1.5 per 1000 live births [11,12]. In our series, CF was diagnosed in 60 prenatal ultrasound anomaly scans (3.72 per 1000 live births). This rate of antenatal diagnosis of CF is relatively elevated. It is probably related to the high percentage of consanguinity in our series (11.7%). Furthermore, the FPR based on the number of fetuses in our series is 6.67%. This rate is considered relatively low in comparison with other studies. By reviewing the literature, this rate varies greatly between research with values of 3.1% [13], 20% [14] or even 40% [8], for example. Table 1 summarizes the association between the accuracy of the diagnosis and several variables. We concluded that the FPR is not associated with the maternal age, the gestational age at the time of the ultrasound, the laterality of CF, the complexity of CF and the fetal gender and presentation. Additionally, none of our cases were labeled prenatally as isolated CF and were reclassified as complex CF in postnatally, as shown in the Bakalis *et al.*’s study, where 19% of the fetuses were not accurately classified [15] or in Lauson *et al.*’s study, where 13% of the fetuses were misclassified [7]. Thus, the accuracy of the diagnosis depends on the standardized approach, the operator’s experience and the quality of the image. The distribution of CF in males to females (7:5) was less than that (2:1) published in several reports [16,17]. In addition, the frequency of bilateral isolated CF was 30%, which is less than that found in Offerdal *et al.*’s study (55%) [13]. We were able to report in our study a significant association between the laterality and the complexity of CF with p-value = 0.003. Furthermore, the frequency of complex CF in our series is 41.7%, which was higher than that found in Bar-On *et al.*’s study (23%) [14] and Di Mascio *et al.*’s study (7.8%) [18]. The percentage of consanguinity may explain this high rate of complex CF. Besides, we did not find in our series a significant association between the amniotic fluid level and the diagnosis of CF, which is comparable with other studies findings [13,19]. In the present study, the amniocentesis was performed in 20 patients (33.3%). Moreover, the prescription of CMA or the standard karyotype is indeed acceptable with complex CF, but it is still debatable in the case of isolated CF. Invasive diagnosis for isolated CF is highly questionable and would be debated in all the vast majority of high-income countries. The frequency of chromosomal defects in patients with isolated CF is denoted as 3.6% in the literature [5]. Singer *et al.* reported that the diagnosis of CF, isolated or complex, increased the risk for abnormal CMA. In our series, amniocentesis was suggested but not performed for some patients. This was due to the nonfinancial coverage of this act or to the decision for pregnancy interruption that was previously taken because of multiple defects. One of 12 (8.3%) of isolated CF that underwent karyotyping revealed a translocation 5–7, while two of eight (25%) of fetuses with complex CF were associated with chromosomal anomalies. Further studies with a larger-sample size could be beneficial to evaluate the efficiency of karyotyping with the prenatal diagnosis of CF. However, 38.3% of the pregnancies included in this study ended with a termination of pregnancy: 14 (23.3%) of the cases ended with medical interruption of pregnancy and nine (15%) ended with stillbirth. We can conclude, based on Table 2, that the consanguinity, the complexity and the bilaterality of CF are risk factors for the termination of pregnancy. On the other hand, we did not find any relationship between the decision of treatment by Ponseti method and the complexity and the laterality of CF. The correlation between the prenatal diagnosis of CF and its severity on postnatal was not studied. Further studies are needed to demonstrate this association, which will improve patient counseling and decrease the morbidity of this defect.

This study has multiple limitations, the main of which include the retrospective data collection. Moreover, the low number of pregnancies that underwent genetic assessment limit the evaluation of the genetic factor on the onset of CF. Although we were able to obtain a FPR of 6.67%, we were unable to provide a false-negative rate. It was not feasible for us to obtain follow up on our entire scanned population. The strengths of our study include the large-size sample and the collection of postnatal outcomes. Compared with other studies where the sample size, for example, was 32 [13], 7 [20], 40 [14], 15 [8], we can consider that we have a relatively large representative sample without any selection or confirmation bias. This present study includes a large, nonselected population, served by the same expert in the same prenatal diagnosis unit, by the same ultrasound machine. Besides the postnatal outcomes, the problem’s management and the parents’ satisfaction level were reported in our study. Thus, prenatal parents counseling on the outcomes of CF had a great impact on their satisfaction postnatally, as they were well prepared, in addition to the presence of a pediatric orthopedic surgeon after birth.

## Conclusion

Clubfoot is one of the most common fetal anomalies detected prenatally on ultrasound. The diagnostic accuracy depends on the standardized approach, the operator’s experience and skills, and the quality of the image. The consanguinity appears to be a risk factor of the interruption of the pregnancy and the complexity of CF. Further studies are needed to evaluate CF during the first trimester and to be able to predict of the severity of CF prenatally.

**Summary points**

**Aim**

- To assess the accuracy of antenatal diagnosis of clubfoot (CF), risk factors, maternal characteristics and fetal outcomes.

**Methods**

- Maternal characteristics, sonographic signs and postnatal results of antenatal diagnosis of CF were evaluated in 60 cases over 16,096 attended live births in a single institution of fetal medicine between 2007 and 2020.

**Results**

- The rate of prenatal diagnosis of CF was 3.72 per 1000 live births. The false-positive rate was 6.67%.
- None of the fetuses was labeled antenatally as isolated CF and diagnosed in postnatal as complex CF.
- The rate of antenatal diagnosis of CF was 3.72/1000 live births. The false-positive rate was 6.67%. 66.7% of fetuses had bilateral CF and 33.3% had unilateral CF, 58.3% were isolated and 41.7% were complex, 58.3% were males and 41.7% were female, 16.7% were multiple pregnancies and 10% were cases of consanguinity.
- In postnatal, 12.5% of infants profited of exclusive physical therapy, while 58% needed Ponseti method and exclusive progressive casting was favorable in 29%.

**Conclusion**

- Consanguinity appears to be a risk factor of the complexity of CF.
- A significant relationship is demonstrated between the interruption of the pregnancy and consanguinity, bilateral CF and complex CF.
